# National Outcomes of Increasing Cervical Cancer Screening in Federally Qualified Health Centers

**DOI:** 10.1001/jamanetworkopen.2025.38593

**Published:** 2025-10-22

**Authors:** Trisha L. Amboree, Prajakta Adsul, Haluk Damgacioglu, Kathleen M. Schmeler, Elizabeth Y. Chiao, Roxana Cruz, Kalyani Sonawane, Ashish A. Deshmukh, Jane R. Montealegre

**Affiliations:** 1 Department of Public Health Science, Medical University of South Carolina, Charleston; 2 Hollings Cancer Center, Medical University of South Carolina, Charleston; 3 Department of Internal Medicine, School of Medicine, University of New Mexico, Albuquerque; 4 Cancer Control and Population Sciences Research Program, University of New Mexico Comprehensive Cancer Center, Albuquerque; 5 Department of Gynecologic Oncology and Reproductive Medicine, The University of Texas MD Anderson Cancer Center, Houston; 6 Departments of Epidemiology and Medical Oncology, The University of Texas MD Anderson Cancer Center, Houston; 7 Texas Association of Community Health Centers, Austin; 8 Department of Behavioral Science, The University of Texas MD Anderson Cancer Center, Houston

## Abstract

**Importance:**

Federally qualified health centers (FQHCs) deliver health care to more than 30 million people across the US and often face structural constraints that affect their ability to provide cervical cancer screening (CCS).

**Objectives:**

To estimate the number of underscreened individuals served by FQHCs and the outcomes of improving CCS in FQHCs to meet the Healthy People 2030 goal of 79.2%.

**Design, Setting, and Participants:**

This cross-sectional analysis used data from the Uniform Data System reported in 2023 by 1352 FQHCs across the US, self-reported survey-weighted CCS prevalence from the National Health Interview Survey, and population size estimates from the US Census Bureau American Community Survey 2023. Participants were US adults served by FQHCs who were CCS eligible nested within the estimated 85 364 685 CCS-eligible individuals in the US population. Analyses were conducted between September 2024 and July 2025.

**Exposures:**

Receipt of CCS at an FQHC.

**Main Outcomes and Measures:**

Percentage of CCS-eligible individuals who were up to date on CCS.

**Results:**

In 2023, a total of 7 757 211 CCS-eligible individuals were served by FQHCs, representing 9.1% (95% uncertainty interval [UI], 9.0%-9.2%) of the estimated 85 364 685 individuals in the CCS-eligible population. FQHCs served 35.9% (95% UI, 32.9%-39.6%) of the underscreened publicly insured population, 26.3% (95% UI, 23.1%-30.1%) of the underscreened rural population, 22.4% (95% UI, 20.9%-24.0%) of the underscreened population living at or below 200% of the poverty level, 19.5% (95% UI, 17.9%-21.4%) of the underscreened uninsured population, and 17.7% (95% UI, 16.7%-18.9%) of the underscreened racial or ethnic minority population. By region, FQHCs served 16.5% (95% UI, 14.6%-18.7%) of the underscreened population in the Northeast, 14.1% (95% UI, 12.7%-15.9%) in the Midwest, 13.5% (95% UI, 12.5%-14.7%) in the South, and 19.1% (95% UI, 17.3%-21.1%) in the West. Increasing CCS in FQHCs from the current estimate of 55.1% to the Healthy People 2030 goal of 79.2% would screen an additional 1 872 367 people and increase CCS among the aforementioned subgroups and regions by 2 to 6 percentage points.

**Conclusions and Relevance:**

This cross-sectional study found that FQHCs served a large number of underscreened individuals from traditionally underscreened subgroups. These results suggest that improving CCS in FQHCs would result in a substantial increase in national CCS rates.

## Key Points

**Questions:**

What proportion of individuals from traditionally underscreened groups is served by federally qualified health centers (FQHCs) and how would increasing cervical cancer screening (CCS) in FQHCs change national screening rates?

**Findings:**

This cross-sectional study of 7 757 211 US adults served by FQHCs found that FQHCs served 35.9% of underscreened publicly insured individuals, 26.3% of underscreened rural individuals, 22.4% of underscreened individuals living at or below 200% of the poverty line, 19.5% of underscreened uninsured individuals, and 17.7% of underscreened individuals from racial or ethnic minority groups. Increasing CCS in FQHCs to the Healthy People 2030 goal (79.2%) would considerably improve national screening rates overall and for each subgroup.

**Meaning:**

These results suggest that initiatives to improve CCS in FQHCs would increase national CCS and mitigate CCS disparities.

## Teaser

This cross-sectional study of US adults investigates what proportion of underscreened individuals are served by federally qualified health centers (FQHCs) and estimates the outcomes of improving cervical cancer screening in FQHCs to meet the Healthy People 2030 goal of 79.2%.

## Introduction

Population-wide implementation of cervical cancer screening (CCS) has led to a greater than 50% reduction in cervical cancer incidence and mortality in the US.^1^ It is projected that with widespread human papillomavirus (HPV) vaccination and high up-to-date (UTD) CCS and treatment of precancer, cervical cancer can be eliminated as a public health problem (defined as <4 cases per 100 000 population) in the US in the next 2 to 3 decades.^2^ However, continued disparities are evident among low-resourced populations, which could significantly delay cervical cancer elimination.^3^ Additionally, previous studies indicate recent increases in cervical cancer incidence and mortality among US women who live in poverty.^4,5^ CCS is notably lower among individuals who are uninsured or publicly insured, are from racial or ethnic minority populations, and live in poverty and in rural areas.^6^ For these populations, barriers to CCS include lack of insurance, time, and transportation; cost and distance to see a health care practitioner; unpaid time taken from work to access health care appointments; competing health care needs; discomfort or inability to undergo a pelvic examination; and psychosocial barriers (eg, distrust of the health care system and low health literacy).^7,8,9,10,11,12^ Perceived cost burden is also a substantial barrier to CCS, including out-of-pocket costs for appointments and future follow-up and treatment costs.^11^

Federally qualified health centers (FQHCs) are a critical component of the US health care safety net,^13^ providing primary care health services at no or low cost on a sliding scale to more than 30 million underserved individuals, including more than 30% of US residents who live at or below the federal poverty line and 20% of US residents who live in rural areas.^14,15^ Funded primarily through Medicaid reimbursement and federal grants, FQHCs provide high-quality, comprehensive care and regularly meet or exceed national primary care benchmarks.^16^ FQHCs nonetheless face multiple and complex challenges to implementing cancer screening services,^17^ including resource constraints created by increasing demand and decreasing per-patient funding,^18^ as well as serving a patient population with a high prevalence of unmet social needs and substantial barriers to care.^17,19^ The confluence of these challenges has resulted in a mean FQHC CCS rate of 51.0% in 2020,^20^ which is well below the national average and the Healthy People 2030 (HP2030) target of 79.2%.^21^

Concerted efforts to improve CCS in FQHCs may significantly improve national UTD CCS. We recently reported that the overall underscreened population served by FQHCs represents a sizeable proportion of the national underscreened general population,^20^ yet this has only been assessed aggregately. Because FQHCs mainly serve subgroups known to have lower CCS rates, improvement in nationally aggregated UTD FQHC CCS could have a substantial impact on national health disparities. In this study, we estimate the number of underscreened individuals who are served by FQHCs across subgroups known to have lower CCS rates and across US geographic regions and examine how national CCS across subpopulations would change if CCS in FQHCs were increased to meet the HP2030 goal of 79.2%.

## Methods

### Data Sources

We used data from the Health Resources and Service Administration (HRSA) Uniform Data System (UDS) 2023^22^ to estimate CCS prevalence in 1352 FQHCs across the contiguous US, Puerto Rico, and the District of Columbia. For CCS prevalence in the general US population, the National Health Interview Survey (NHIS) 2021^23^ was used to estimate population-weighted CCS uptake and hysterectomy prevalence, and the US Census Bureau American Community Survey 2023 1-Year Estimate data^24^ were used to estimate the US population size and derive the CCS-eligible population size in the general US population. This study followed the Strengthening the Reporting of Observational Studies in Epidemiology (STROBE) reporting guideline for cross-sectional studies and was deemed exempt from approval and informed consent by the institutional review board at Medical University of South Carolina because the data are deidentified and publicly available.

### Main Outcome Measures

The US Preventive Services Task Force guidelines^25^ were used to determine CCS prevalence. For FQHC data, persons aged 21 to 64 years with a cervix were considered CCS eligible. UTD CCS was defined as having cervical cytologic screening (ie, Papanicolaou test) within 3 years for individuals aged 21 to 64 years or having high-risk HPV testing performed within 5 years for individuals aged 30 to 64 years. The UDS measures exclude individuals who had a complete hysterectomy and individuals who were in hospice care during the measurement period. To estimate CCS prevalence in the general US population, individuals aged 21 to 65 years with a cervix were considered CCS eligible. Using NHIS data, we defined population-weighted UTD CCS prevalence as having cervical cytologic screening performed within 3 years for individuals aged 21 to 65 years or having high-risk HPV testing (alone or with cervical cytologic screening) performed within 5 years for individuals aged 30 to 65 years. Individuals with a history of hysterectomy were excluded.

All findings are presented overall, by demographic subgroup, and by geographic region. Subgroups assessed were those reported in the literature to have lower CCS rates^6^ and include populations who are publicly insured (reference: privately insured), uninsured (reference: insured), those living at or below 200% of the federal poverty level (a commonly used measure to define low income^26^; reference: those living above 200% of the federal poverty level), those from racial or ethnic minority groups, which included American Indian or Alaska Native, Asian, Black or African American, Hispanic (all races), Native Hawaiian or Pacific Islander, or multiple races (reference: non-Hispanic White), and those from rural areas (reference: urban). Geographic regions were based on designations in the NHIS and US Census Bureau data and include Northeast (Connecticut, Maine, Massachusetts, New Hampshire, Rhode Island, Vermont, New Jersey, New York, and Pennsylvania), Midwest (Illinois, Indiana, Michigan, Ohio, Wisconsin, Iowa, Kansas, Minnesota, Missouri, Nebraska, North Dakota, and South Dakota), South (Delaware, District of Columbia, Florida, Georgia, Maryland, North Carolina, South Carolina, Virginia, West Virginia, Alabama, Kentucky, Mississippi, Tennessee, Arkansas, Louisiana, Oklahoma, and Texas), and West (Arizona, Colorado, Idaho, Montana, Nevada, New Mexico, Utah, Wyoming, Alaska, California, Hawaii, Oregon, and Washington). Puerto Rico was not included in regional analyses.

### Statistical Analysis

Cross-sectional analyses were conducted between September 2024 and July 2025 using SAS software, version 9.4 (SAS Institute Inc) and R, version 4.4.1 (R Foundation for Statistical Computing). For national UTD CCS in FQHCs and overall, percentages and binomial percentage 2-sided 95% CIs are presented. For the general US population, the overall and subgroup- or regional-level CCS-eligible populations were calculated by (1) estimating the NHIS population-weighted hysterectomy prevalence overall and for each group, (2) multiplying the hysterectomy prevalence by the total census population for age-eligible female individuals to estimate the number of those who had a hysterectomy overall and for each group, and (3) subtracting the number of those who had a hysterectomy from the total census population size of age-eligible female individuals overall and for each subgroup. On the basis of the calculated numbers, we estimated the proportion of the CCS-eligible population served by FQHCs by dividing the number of FQHC CCS-eligible population by the estimated number of the CCS-eligible population within the general US population overall and for each subgroup and region. Next, the overall and subgroup- or regional-level UTD CCS rates were calculated by multiplying the CCS-eligible population by the population-weighted CCS prevalence derived from the NHIS data. Individuals were considered underscreened if they were CCS eligible but not UTD. Then, we estimated the proportion of the underscreened population served by FQHCs by dividing the number of FQHC underscreened individuals by the estimated number of underscreened individuals within the general US population overall and for each subgroup and region (eMethods, eFigure 1, and eFigure 2 in Supplement 1).

We calculated the percentage point difference in CCS overall and for each subgroup in the general US population and across each geographic region that would be attained under the hypothetical scenario of meeting the HP2030 CCS goal of 79.2% in FQHCs. To generate these scenarios, we (1) estimated the number of CCS-eligible persons served in FQHCs overall and for each subgroup or region from UDS data, (2) multiplied the number of CCS-eligible persons served in FQHCs by 0.792 to calculate the number of persons screened in FQHCs if meeting HP2030 goals overall and for each subgroup and region, (3) subtracted the observed number of persons screened in FQHCs from the hypothetical number of persons screened in FQHCs if meeting HP2030 goals to quantify the difference in persons screened in FQHCs overall and for each subgroup and region, (4) added the difference in persons screened in FQHCs after meeting HP2030 goals to the number of observed CCS-eligible persons screened in the general population, and (5) divided the hypothetical number of CCS-eligible persons screened in the general population after meeting HP2030 goals in FQHCs by the CCS-eligible population to estimate the hypothetical proportion of UTD CCS after improving screening to meet the HP2030 goal in FQHCs. Based on this estimate, we calculated the percentage point change in screening in the general population if screening uptake was improved in FQHCs to meet the HP2030 goal. Given that the data for the proportions of CCS-eligible and underscreened populations served by FQHCs, as well as differences in screening after improvements in FQHCs, are from 3 different data sources (eg, FQHC data, NHIS data, and US Census data), we assumed a binomial distribution for FQHC data and a normal distribution for NHIS data, then calculated 95% uncertainty intervals (95% UIs) using 1000 Monte Carlo resampling simulations to account for this difference. All statistical testing (ie, the calculation of confidence intervals and uncertainty intervals) was set as 2-sided α level of .05.

## Results

**Figure 1.**
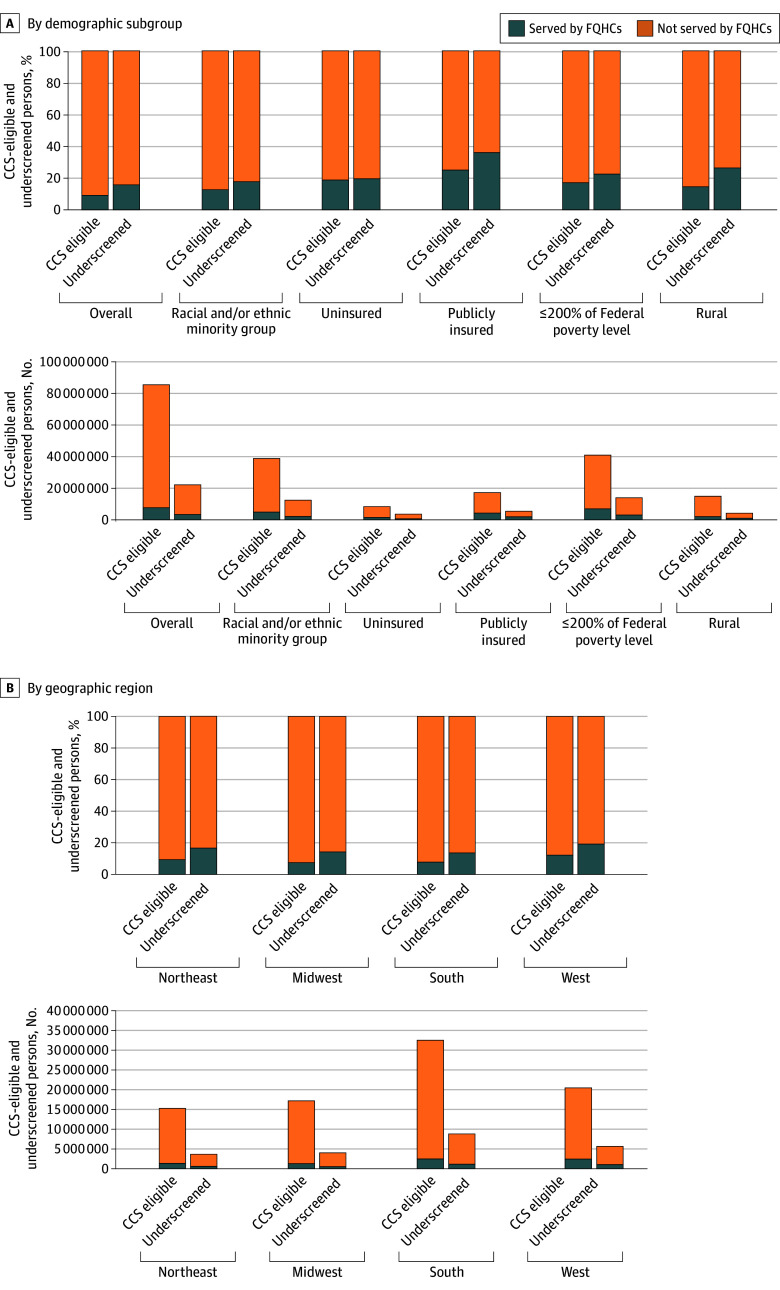
Proportion and Number of Cervical Cancer Screening (CCS)–Eligible and Underscreened Individuals Served by Federally Qualified Health Centers (FQHCs) by Demographic Subgroup and Geographic Region Racial and/or ethnic minority groups included American Indian or Alaska Native, Asian, Black or African American, Hispanic (all races), Native Hawaiian or Pacific Islander, or multiple races.

In 2023, a total of 7 757 211 CCS-eligible individuals were served by FQHCs, representing 9.1% (95% UI, 9.0%-9.2%) of the overall US CCS-eligible population, estimated to be 85 364 685 individuals (Figure 1A, Table 1). When stratified by demographic subgroups, FQHCs served 18.8% (95% UI, 18.4%-19.2%) of the CCS-eligible uninsured population (1 600 210 of 8 518 681), 14.5% (95% UI, 14.0%-14.9%) of the CCS-eligible rural population (2 170 794 of 14 982 999), 17.0% (95% UI, 16.8%-17.3%) of the CCS-eligible population living at or below 200% of the poverty level (6 970 990 of 40 958 868), 12.7% (95% UI, 12.5%-12.8%) of the CCS-eligible population from racial or ethnic minority groups (4 914 431 of 38 829 285), and 24.9% (95% UI, 24.5%-25.4%) of the CCS-eligible publicly insured population (4 331 323 of 17 369 415) (Figure 1A, Table 1). When stratified by region, FQHCs served 9.3% (95% UI, 9.2%-9.4%) of the CCS-eligible population in the Northeast, 7.4% (95% UI, 7.3%-7.6%) in the Midwest, 7.7% (95% UI, 7.5%-7.8%) in the South, and 12.1% (95% UI, 11.9%-12.3%) in the West (Figure 1A, Table 1).

**Table 1.** CCS-Eligible and Underscreened Individuals Served by FQHCs

Subgroup	CCS-eligible individuals			Underscreened		
	FQHCs, No.	Overall, No.	Served in FQHCs, % (95% UI)	FQHCs, No.	Overall, No.	Served in FQHCs, % (95% UI)
All	7 757 211	85 364 685	9.1 (9.0-9.2)	3 485 867	22 171 257	15.7 (15.0-16.6)
Rural	2 170 794	14 982 999	14.5 (14.0-14.9)	1 107 683	4 210 343	26.3 (23.1-30.1)
Racial and/or ethnic minority group^a^	4 914 431	38 829 285	12.7 (12.5-12.8)	2 208 404	12 486 217	17.7 (16.7-18.9)
Uninsured	1 600 210	8 518 681	18.8 (18.4-19.2)	719 075	3 693 513	19.5 (17.9-21.4)
Publicly insured	4 331 323	17 369 415	24.9 (24.5-25.4)	1 946 336	5 420 004	35.9 (32.9-39.6)
≤200% of Federal poverty level	6 970 990	40 958 868	17.0 (16.8-17.3)	3 132 562	13 967 711	22.4 (20.9-24.0)
Region						
Northeast	1 418 882	15 263 188	9.3 (9.2-9.4)	604 199	3 661 883	16.5 (14.6-18.7)
Midwest	1 273 415	17 147 541	7.4 (7.3-7.6)	567 559	4 037 423	14.1 (12.7-15.9)
South	2 491 341	32 519 527	7.7 (7.5-7.8)	1 188 972	8 829 670	13.5 (12.5-14.7)
West	2 469 544	20 438 812	12.1 (11.9-12.3)	1 079 808	5 653 089	19.1 (17.3-21.1)

Abbreviations: CCS, cervical cancer screening; FQHC, federally qualified health center; UI, uncertainty interval.

^a^ Racial and/or ethnic minority groups included American Indian or Alaska Native, Asian, Black or African American, Hispanic (all races), Native Hawaiian or Pacific Islander, or multiple races.

The proportion of the national underscreened population served by FQHCs was 15.7% (95% UI, 15.0%-16.6%) (3 485 867 of 22 171 257). When stratified by demographic subgroups, FQHCs served 19.5% (95% UI, 17.9%-21.4%) of the underscreened uninsured population (719 075 of 3 693 513), 35.9% (95% UI, 32.9%-39.6%) of the underscreened publicly insured population (1 946 336 of 5 420 004), 17.7% (95% UI, 16.7%-18.9%) of the underscreened population from racial or ethnic minority groups (2 208 404 of 12 486 217), 22.4% (95% UI, 20.9%-24.0%) of the underscreened population living at or below 200% of the poverty level (3 132 562 of 13 967 711), and 26.3% (95% UI, 23.1%-30.1%) of the underscreened rural population (1 107 683 of 4 210 343) (Figure 1A, Table 1). When stratified by region, FQHCs served 16.5% (95% UI, 14.6%-18.7%) of the underscreened population in the Northeast, 14.1% (95% UI, 12.7%-15.9%) in the Midwest, 13.5% (95% UI, 12.5%-14.7%) in the South, and 19.1% (95% UI, 17.3%-21.1%) in the West (Figure 1B, Table 1).

**Figure 2.**
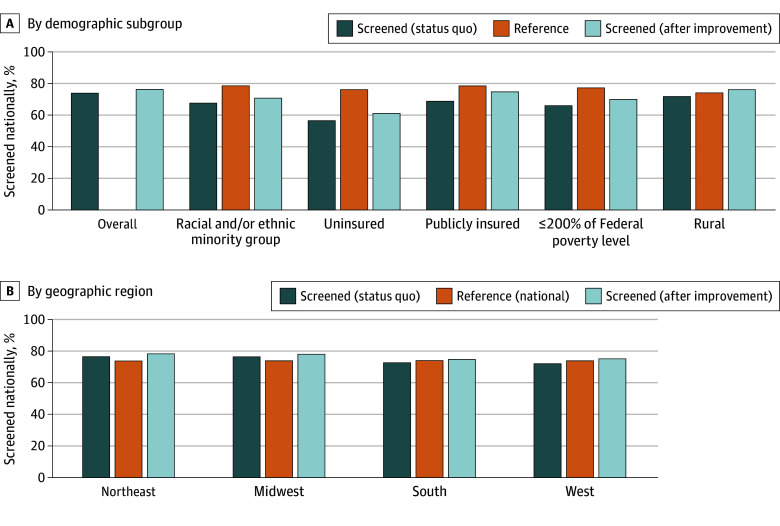
Estimated Outcomes of Improving Screening in Federally Qualified Heath Centers on National Cervical Cancer Screening Rates by Demographic Subgroup and Geographic Region A, Reference groups for the subgroups were non-Hispanic White, insured (inclusive of public insurance), privately insured, above the 200% federal poverty level and urban, respectively. B, Reference group for regions was the overall national cervical cancer screening estimate (74.0%).

Nationally, the UTD CCS rate was 55.1% (95% CI, 55.0%-55.1%) in FQHCs and 74.0% (95% CI, 72.7%-75.3%) in the general US population. The estimated potential change in national CCS disparities by improving UTD CCS in FQHCs is detailed in Figure 2 and Table 2. Improving CCS in FQHCs to the HP2030 goal of 79.2% would result in an estimated additional 1.87 million people screened and increase the overall national UTD CCS by 2.2 (95% UI, 1.5-3.0) percentage points (from 74.0% to 76.2%). By subgroup, this would increase UTD CCS by 4.5 (95% UI, 2.6-6.6) percentage points (from 56.6% to 61.2%) among the uninsured (additional 386 000 screened), 6.0 (95% UI, 4.4-7.6) percentage points (from 68.8% to 74.8%) among the publicly insured (additional 1.05 million screened), 4.1 (95% UI, 2.9-5.4) percentage points (from 65.9% to 70.0%) among people living at or below 200% of the poverty level (additional 1.68 million screened), 3.1 (95% UI, 2.0-4.1) percentage points (from 67.8% to 70.9%) among those from racial and ethnic minority groups (additional 1.19 million screened), and 4.4 (95% UI, 2.0-6.8) percentage points (from 71.9% to 76.3%) among rural residents (additional 656 000 screened) (Figure 2A, Table 2). Across US regions, improving CCS in FQHCs to the HP2030 goal of 79.2% would increase UTD CCS by 2.0 (95% UI, 0.7-3.4) percentage points (from 76.0% to 78.0%) in the Northeast (additional 309 000 screened), 1.8 (95% UI, 0.1-3.5) percentage points (from 76.5% to 78.2%) in the Midwest (additional 303 000 screened), 2.1 (95% UI, 0.7-3.4) percentage points (from 72.8% to 74.9%) in the South (additional 671 000 screened), and 2.8 (95% UI, 1.4-4.1) percentage points (from 72.3% to 75.1%) in the West (additional 566 000 screened) (Figure 2B, Table 2).

**Table 2.** Estimated Outcomes of Improving Screening in Federally Qualified Health Centers on National Cervical Cancer Screening

Subgroup^a^	Population, % (95% CI)			Percentage point increase in screened (95% UI)	Increase in screened, No. (95% UI)
	Screened (status quo)	Reference	Screened (after improvement)		
Overall	74.0 (72.7-75.3)	NA	76.2 (75.5-76.9)	2.2 (1.5-3.0)	1 872 367 (1 255 829-2 503 321)
Racial and/or ethnic minority group	67.8 (65.8-69.8)	78.7 (77.2-80.2)	70.9 (70.1-71.7)	3.1 (2.0-6.8)	1 186 202 (767 146-1 610 107)
Uninsured	56.6 (52.6-60.7)	76.2 (75.0-77.5)	61.2 (60.0-62.5)	4.5 (2.6-6.6)	386 231 (206 576-560 309)
Publicly insured	68.8 (65.9-71.7)	78.6 (77.2-79.9)	74.8 (73.5-76.3)	6.0 (4.4-7.6)	1 045 421 (766 597-1 327 638)
≤200% of Federal poverty level	65.9 (63.6-68.2)	77.5 (76.1-78.8)	70.0 (69.0-71.1)	4.1 (2.9-5.4)	1 682 596 (1 149 685-2 174 653)
Rural	71.9 (68.3-75.5)	74.3 (72.9-75.7)	76.3 (73.9-78.9)	4.4 (2.0-6.8)	656 158 (274 486-1 041 013)
Region^b^					
Northeast	76.0 (72.9-79.1)	74.0 (72.7-75.3)	78.0 (76.9-79.3)	2.0 (0.7-3.4)	309 072 (107 960-541 449)
Midwest	76.5 (73.8-79.1)	74.0 (72.7-75.3)	78.2 (76.9-79.6)	1.8 (0.1-3.5)	302 689 (13 697-579 391)
South	72.8 (70.8-74.9)	74.0 (72.7-75.3)	74.9 (73.8-76.1)	2.1 (0.7-3.4)	670 773 (234 458-1 140 491)
West	72.3 (69.6-75.1)	74.0 (72.7-75.3)	75.1 (74.0-76.3)	2.8 (1.4-4.1)	566 143 (288 694-823 399)

Abbreviations: NA, not applicable; UI, uncertainty interval.

^a^ Reference groups for subgroups were non-Hispanic White, insured (inclusive of public insurance), privately insured, above 200% federal poverty level, and urban, respectively.

^b^ Reference group for regions was the overall national cervical cancer screening estimate (74.0%).

## Discussion

Findings from this study indicate that FQHCs across US states, Puerto Rico, and the District of Columbia serve 9.1% of the 85.4 million individuals who are eligible for CCS but have a UTD CCS rate of only 55.1%. Reflecting this CCS rate and the composition of the FQHC population, FQHCs serve 19.5% of underscreened individuals who are uninsured, 22.4% who are living at or below 200% of the federal poverty level, 17.7% who are from racial or ethnic minority populations, 26.3% of underscreened individuals who live in rural areas, and 35.9% of underscreened individuals who are publicly insured. Thus, a substantial portion of the gap between current UTD CCS rates and the HP2030 goal is concentrated within the FQHC population. Targeted interventions within FQHCs represent an opportunity to allocate resources that can reduce this disparity. Our analysis estimates that such interventions could result in an additional 1.87 million CCS-eligible persons being screened, translating to an increase of 2.2 percentage points in the national screening rate. Given the demographic composition of the FQHC population, these gains would be particularly meaningful among groups with traditionally low screening rates. Specifically, we estimated increases in UTD screening from 68.8% to 74.8% among publicly insured individuals, from 56.6% to 61.2% among the uninsured, from 71.9% to 76.3% among rural residents, and from 65.9% to 70.0% among individuals living at or below 200% of the federal poverty level. Although these improvements alone may not achieve the HP2030 target, strategic investment in preventive services at FQHCs could substantially advance progress, particularly for underserved populations. Additionally, improvements in CCS within FQHCs could meaningfully enhance regional screening rates, particularly in the South and West, where CCS coverage is lowest and the number of CCS-eligible individuals is highest.

Achieving substantive improvements in UTD CCS in FQHCs has historically been challenging. Most CCS in the US involves cytologic testing either alone or with cotesting for high-risk HPV.^27,28^ This poses a significant challenge because cytologic testing and cotesting require that a pelvic examination be performed by a health care practitioner. Doing so for all CCS-eligible patients is challenged by capacity constraints, cost, competing health care needs, and patients’ unwillingness and/or inability to undergo a pelvic examination.^8,12,17^ Primary high-risk HPV testing with self-collection, whereby individuals can take their own vaginal sample, offers a new, effective screening option for underscreened individuals who have been unable or unwilling to be screened by a health care practitioner.^29,30^ Data from the US and international settings indicate high acceptability and a 2-fold improvement in UTD CCS when offering self-collection vs practitioner-performed CCS.^29,30,31^ New evidence indicates that self-collection is particularly effective in safety net health settings.^31^ With the approval for use in health care settings by the US Food and Drug Administration in 2024, self-collection can now be integrated into clinical workflows to address many of the challenges posed by practitioner-performed CCS. Given this new option, FQHCs may consider integrating self-collection in primary care settings to facilitate opportunistic, same-visit CCS among patients visiting FQHCs for unrelated health needs and/or to circumvent the need for a pelvic examination.

In addition to self-collection, enabling services offered by FQHCs to address barriers to care, which include patient education and patient navigation, are known to improve use of preventive care services^32,33^ and can act synergistically with self-collection to increase CCS.^31^ Cancer prevention initiatives supported by the HRSA, such as the one launched in 2024,^34^ are critical for building CCS capacity and could be leveraged to provide technical assistance and funding for services to accelerate the broad adoption and implementation of self-collection in FQHCs.^34^ Collectively, investment in the integration of self-collection and the provision of enabling services could provide a tangible way to address low CCS rates in FQHCs. However, sustained investment in FQHCs may be affected by evolving health care policies and changes in funding streams, including support for Medicaid and the HRSA. Reductions in these areas could further limit available resources for FQHCs, potentially impacting their capacity to implement strategies aimed at improving cancer screening rates.^35^ Notably, a recent study projected that funding reductions to Medicaid could result in approximately 775 691 additional CCS-eligible individuals who may not receive recommended CCS.^35^

Methodologically, our analysis explores a novel method to estimate the national screen-eligible population size overall and among subgroups known to have lower CCS rates, thereby explicitly highlighting opportunities to mitigate cancer screening disparities. Survey-weighted estimates are appropriately positioned to estimate the prevalence of screening behaviors^36^ but may not be the proper tool to derive screen-eligible population sizes. Thus, extrapolating NHIS behavioral prevalence estimates onto population size estimates from the US Census help to provide a clearer picture of national screening disparities and the impact of targeted improvements. In the absence of a national cancer screening registry, data from multiple sources are needed to fill this gap.

### Limitations

Our results should be interpreted considering the study’s limitations. First, each dataset has its own inherent biases (ie, NHIS is individual-level, self-reported, survey-weighted data; UDS is clinic-level, practice-based data; and the US Census data are based on population estimates). We present estimates with 95% UIs to account for the potential bias introduced from using these distinct databases. Furthermore, the use of American Community Survey data to estimate the population size of demographic subgroups who are CCS eligible may be limited due to the nature of who responds to US Census surveys. Second, we assumed that the proportion of screened persons in FQHCs was consistent across subgroups (except for rural estimates and geographic regions, which we were able to derive directly) to estimate the number of CCS-eligible and screened persons served by FQHCs within each subgroup. Third, we did not use NHIS 2023 data because questions about cervical cancer screening modality were not included in this data year. Given that recommended screening time frames differ based on modality, we used NHIS 2021 data because these were the most recent data with this information available. Fourth, HPV vaccination is a critical component in cervical cancer prevention; however, data on HPV vaccination of FQHC patients are not available and thus not included in this analysis. Nevertheless, practice-based data help provide an indication of missed opportunities to reduce CCS disparities, and census data help contextualize these findings within the broader US population.

## Conclusions

In this cross-sectional study, our data show that concerted efforts to implement evidence-based strategies to improve CCS rates in FQHCs to meet the HP2030 goal of 79.2% could substantially increase national UTD CCS and address historic and continuing CCS disparities. Initiatives focused on improving screening, such as the implementation of HPV self-collection in FQHCs, should be prioritized to increase CCS rates and move closer to mitigating CCS disparities.

## Notes

Supplement 1. **eMethods.** Overview of Participant Identification Flow to Estimate CCS-Eligible and Screened Population in US FQHCS and the General US Population **eFigure 1.** Simplified Schematic of Participant Identification Flow **eFigure 2.** Participant Identification Flow Example Using Publicly Insured Population

Supplement 2.
Data Sharing Statement
