# Supplementary material for: National Outcomes of Increasing Cervical Cancer Screening in Federally Qualified Health Centers
Source: JAMA Netw Open. 2025 Oct 22;8(10):e2538593. doi: 10.1001/jamanetworkopen.2025.38593 (PMC12547585; doi:10.1001/jamanetworkopen.2025.38593)
Supplement: Supplement 1. — eMethods. Overview of Participant Identification Flow to Estimate CCS-Eligible and Screened Population in US FQHCS and the General US Population eFigure 1. Simplified Schematic of Participant Identification Flow eFigure 2. Participant Identification Flow Example Using Publicly Insured Population [file jamanetwopen-e2538593-s001.pdf]

Supplemental Online Content

Amboree TL, Adsul P, Damgacioglu H, et al. National outcomes of increasing cervical cancer screening in federally qualified health centers. *JAMA Netw Open*. 2025;8(10):e2538593. doi:10.1001/jamanetworkopen.2025.38593

eMethods. Overview of Participant Identification Flow to Estimate CCS-Eligible and Screened Population in US FQHCS and the General US Population
eFigure 1. Simplified Schematic of Participant Identification Flow
eFigure 2. Participant Identification Flow Example Using Publicly Insured Population

This supplemental material has been provided by the authors to give readers additional information about their work.

© 2025 Amboree TL et al. *JAMA Network Open*.

eMethods. Overview of participant identification flow to estimate CCS-eligible and screened population in US FQHCs and the general US population.

We generated scenarios for the national estimated outcomes of increasing the cervical cancer screening uptake in federally qualified health centers (FQHCs). To do this, we used data from the Uniform Data System (UDS) Health Resources and Services Administration (HRSA) to estimate the FQHC screen-eligible and screened populations overall and by demographic subgroup (simplified schematic in **Supplement Figure 1A** and **Supplement Figure 2A**) and the US Census and National Health Interview Survey (NHIS) to estimate the screen-eligible and screened populations in the general US population overall and by subgroup (simplified schematic in **Supplement Figure 1B** and **Supplement Figure 2B**). With data provided by each data source, we estimated the number of screen-eligible individuals overall and by demographic subgroup, along with the number of screened individuals overall and by demographic subgroups. With the exception of rural classifications (which are directly reported in the UDS HRSA data for each FQHC system), we assumed the screen-eligible proportion of individuals for each demographic subgroup served by FQHCs was the same as the overall proportion of individuals served by FQHCs for that subgroup. We also assumed that screening uptake was consistent across all demographic subgroups served by FQHCs (e.g., 55.1%). We generated the scenarios for 79.2% screening use in FQHCs to meet Healthy People 2030 goals. The following steps were used for each cohort:

© 2025 Amboree TL et al. *JAMA Network Open*.

eFigure 1. Simplified schematic of participant identification flow.

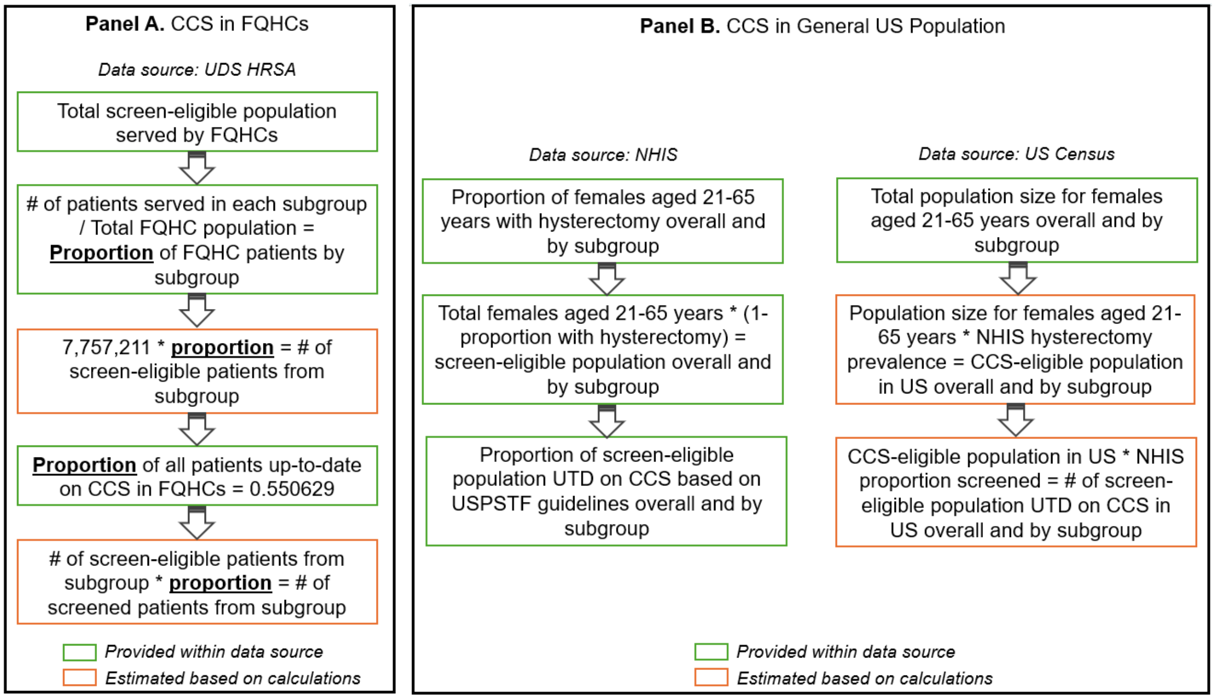

Abbreviations: FQHC, federally qualified health center; CCS, cervical cancer screening; US, United States; UDS HRSA, Uniform Data System Health Resources and Services Administration; NHIS, National Health Interview Survey; UTD, up-to-date

© 2025 Amboree TL et al. *JAMA Network Open*.

eFigure 2. Participant identification flow example using publicly insured population.

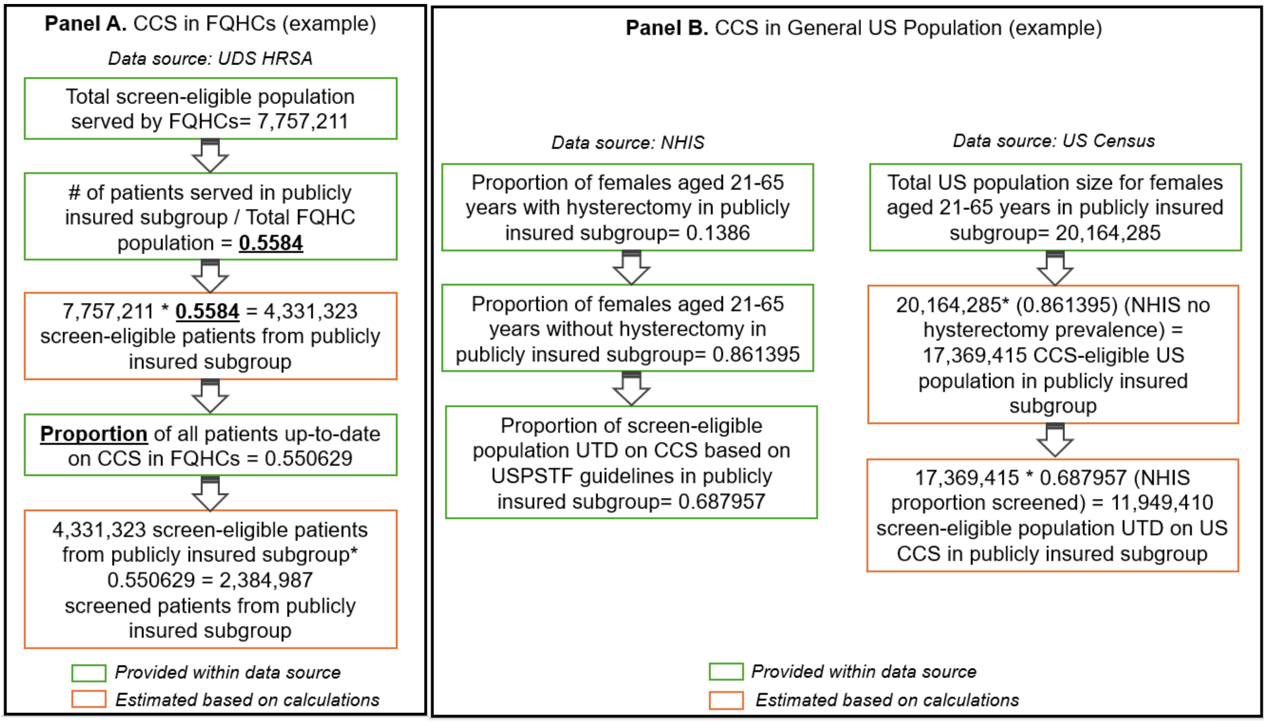

Abbreviations: FQHC, federally qualified health center; CCS, cervical cancer screening; US, United States; UDS HRSA, Uniform Data System Health Resources and Services Administration; NHIS, National Health Interview Survey; UTD, up-to-date

© 2025 Amboree TL et al. *JAMA Network Open*.
