# Supplementary material for: National Outcomes of Increasing Cervical Cancer Screening in Federally Qualified Health Centers
Source: JAMA Netw Open. 2025 Oct 22;8(10):e2538593. doi: 10.1001/jamanetworkopen.2025.38593 (PMC12547585; doi:10.1001/jamanetworkopen.2025.38593)
Supplement: Supplement 2. — Data Sharing Statement [file jamanetwopen-e2538593-s002.pdf]

Data Sharing Statement

Amboree. National Outcomes of Increasing Cervical Cancer Screening in Federally Qualified Health Centers. *JAMA Netw Open*. Published October 22, 2025. doi:10.1001/jamanetworkopen.2025.38593

Data
Data available: No

Additional Information
Explanation for why data not available: Data for the Health Resources and Services Administration's (HRSA) Uniform Data System (UDS) 2023, National Health Interview Survey (NHIS) 2021, and US Census Bureau American Community Survey are publicly available at www.hrsa.gov/foia/electronic-reading, https://www.cdc.gov/nchs/nhis/documentation/20210-nhis.html, and https://data.census.gov/table, respectively.
